# A Highly Stable Electrochemical Sensor Based on a Metal–Organic Framework/Reduced Graphene Oxide Composite for Monitoring the Ammonium in Sweat

**DOI:** 10.3390/bios14120617

**Published:** 2024-12-15

**Authors:** Yunzhi Hua, Junhao Mai, Rourou Su, Chengwei Ma, Jiayi Liu, Cong Zhao, Qian Zhang, Changrui Liao, Yiping Wang

**Affiliations:** 1 School of Information and Communication, Shenzhen Institute of Information Technology, Shenzhen 518172, China; huayz@sziit.edu.cn; 2 Guangdong Laboratory of Artificial Intelligence and Digital Economy (SZ), Shenzhen 518107, China; 2210273110@email.szu.edu.cn (J.M.); surourou2019@email.szu.edu.cn (R.S.); 2310543014@email.szu.edu.cn (C.M.); 2410813004@mails.szu.edu.cn (J.L.); ypwang@szu.edu.cn (Y.W.); 3 Key Laboratory of Clinical Evaluation Technology for Medical Device of Zhejiang Province, The First Affiliated Hospital, Zhejiang University School of Medicine, Hangzhou 310009, China; 1317070@zju.edu.cn; 4 Key Laboratory of Optoelectronic Devices and Systems of Ministry of Education and Guangdong Province, College of Physics and Optoelectronic Engineering, Shenzhen University, Shenzhen 518060, China; cliao@szu.edu.cn; 5 Shenzhen Key Laboratory of Photonic Devices and Sensing Systems for Internet of Things, Guangdong and Hong Kong Joint Research Centre for Optical Fiber Sensors, Shenzhen University, Shenzhen 518060, China

**Keywords:** wearable sensor, ammonium ion detection, metal–organic framework (MOF), reduced graphene oxide (rGO), sweat monitoring

## Abstract

The demand for non-invasive, real-time health monitoring has driven advancements in wearable sensors for tracking biomarkers in sweat. Ammonium ions (NH_4_^+^) in sweat serve as indicators of metabolic function, muscle fatigue, and kidney health. Although current ion-selective all-solid-state printed sensors based on nanocomposites typically exhibit good sensitivity (~50 mV/log [NH_4_^+^]), low detection limits (LOD ranging from 10^−6^ to 10^−7^ M), and wide linearity ranges (from 10^−5^ to 10^−1^ M), few have reported the stability test results necessary for their integration into commercial products for future practical applications. This study presents a highly stable, wearable electrochemical sensor based on a composite of metal–organic frameworks (MOFs) and reduced graphene oxide (rGO) for monitoring NH_4_^+^ in sweat. The synergistic properties of Ni-based MOFs and rGO enhance the sensor’s electrochemical performance by improving charge transfer rates and expanding the electroactive surface area. The MOF/rGO sensor demonstrates high sensitivity, with a Nernstian response of 59.2 ± 1.5 mV/log [NH_4_^+^], an LOD of 10^−6.37^ M, and a linearity range of 10^−6^ to 10^−1^ M. Additionally, the hydrophobic nature of the MOF/rGO composite prevents water layer formation at the sensing interface, thereby enhancing long-term stability, while its high double-layer capacitance minimizes potential drift (7.2 µV/s (i = ±1 nA)) in short-term measurements. Extensive testing verified the sensor’s exceptional stability, maintaining consistent performance and stable responses across varying NH_4_^+^ concentrations over 7 days under ambient conditions. On-body tests further confirmed the sensor’s suitability for the continuous monitoring of NH_4_^+^ levels during physical activities. Further investigations are required to fully elucidate the impact of interference from other sweat components (such as K^+^, Na^+^, Ca^2+^, etc.) and the influence of environmental factors (including the subject’s physical activity, posture, etc.). With a clearer understanding of these factors, the sensor has the potential to emerge as a promising tool for wearable health monitoring applications.

## 1. Introduction

The development of non-invasive, real-time monitoring systems for health and fitness has gained significant traction in recent years. Among these, wearable sensors designed to monitor biochemical markers in human sweat, as well as other biofluids such as tears and saliva, offer a promising method for continuous health tracking [1]. Sweat is an accessible biofluid, and its composition, including electrolytes, metabolites, and other analytes, reflects physiological changes that can be monitored to assess health conditions such as dehydration, stress, and metabolic disorders [2]. Similarly, other biofluids like tears and saliva also contain biomarkers that can provide valuable insights into health status, making them potential candidates for similar sensor applications.

The concentration of ammonium ions (NH_4_^+^) in sweat has gained attention as a potential indicator of muscle fatigue, metabolic efficiency, and kidney function [3]. Typically, this concentration is monitored by ion-selective electrode (ISE)-based wearable electrochemical sensors [4]. Ammonium ion-selective electrodes (NH_4_^+^-ISE) are electrochemical sensors that convert the activity of the selected ion NH_4_^+^, in a sample solution, into a potential output (for the electrochemical mechanism of NH_4_^+^ detection and its comparison with that of metal-oxide thin film pH sensors, please refer to the Supplementary Material). Ammonium ion concentrations in human sweat can be as low as 10^−4^ M, while other cations, such as Ca^2+^, can be as low as 7 × 10^−6^ M [5]. Thus, creating a sensor that is both highly sensitive and reliable while also being flexible and durable remains challenging. Thus, potential stability is a critical performance metric, with both short-term and long-term stability being especially important. Long-term stability is assessed by systematically measuring the sensor’s potential at regular intervals over extended periods (e.g., days, weeks, or months), often characterized by the change in potential over time [6]. Some researchers have highlighted that sensor performance and long-term stability—extending to several months or more—are critical for commercial viability [7]. Short-term stability, on the other hand, can be quickly evaluated using chronopotentiometry (CP), by measuring the change in electrode potential over a short period (e.g., 60 s) after applying a brief electrical impulse (typically in nA) to the electrode [8]. The potential drift, indicative of stability, is then determined from a linear segment of the chronopotentiometric curve using the dE/dt relationship.

A common approach to enhance electrode stability is to coat the electrode, made of a stable and inert metal, with a protective porous membrane typically composed of polyvinyl butyral (PVB) [9], which also allows for the free exchange of ions. Additionally, both electrode nano-structuration and the deposition of polymers exhibit the positive effect of reducing the potential drift, which also enhance the stability of the sensor. Nanomaterials used in electrode modification can generally be categorized into three types: carbon nanomaterials, metal and metal oxide nanoparticles, and nanocomposites [10].

By utilizing these nanocomposites, the ion-selective all-solid-state printed electrochemical sensors for NH_4_^+^ detection have demonstrated excellent sensitivity, a limit of detection (LOD), and linearity in in situ analyses of environmental water samples [11,12,13]. Coutinho et al. used SiO_2_/ZrO_2_/Phosphate- NH_4_^+^ as an ionophore and graphite powder as a solid contact to fabricate an ammonium ion sensor for the quantification of ammonium ions in natural waters, which achieved a low LOD of 1.6 × 10^−7^ M, although the preparation of the composite was quite time-consuming [14]. More recently, other sensors have been developed using nonactin-based ion-selective membranes (ISM) but with different types of nanocomposites as the solid contact, such as CNT-PVC composites (composite polyvinyl chloride membrane impregnated with carbon nanotubes) [11], MMA-DMA copolymers (plasticizer-free methyl methacrylate–decyl methacrylate copolymer) [11], CPANI (copolymer of aniline/2,5-dimethoxyaniline) [12], and graphite–PVB [13] composites. These sensors typically achieve sensitivities higher than 50 mV/log [NH_4_^+^], with LODs ranging from 10^−6^ to 10^−7^ M and linearity ranges from 10^−5^ to 10^−1^ M. Additionally, we have developed a highly stretchable electrochemical sensor based on a 3D Graphene Oxide–CNT composite, maintaining high sensitivity of 59.2 mV/log [NH_4_^+^] even under a strain of 40% [15]. However, stability testing results for these sensors are still limited. Only Athavale et al. have reported that the CNT-PVC composite-based sensor exhibited a short-term potential drift of <1000 μV/h (i = 0), while the MMA-DMA copolymer-based sensor exhibited a short-term potential drift of 3600 μV/h (i = 0) [11]. Therefore, the development of wearable sensors with enhanced potential stability remains essential, particularly for reliable ammonium ion monitoring in sweat.

Metal–organic frameworks (MOFs) represent a novel class of materials for sensor development. Due to their high porosity and large surface areas, MOFs enhance interactions with analytes, significantly improving sensor sensitivity. Conductive MOFs, in particular, also contribute to improved stability [16]. Conductive MOFs were utilized as supercapacitors, enabling sensors with high capacitance (204 μF) and excellent potential stability (drift of 11.1 A μh^−1^), while also preventing the formation of a water layer on the electrodes [17]. Among MOFs, Ni-based frameworks have exhibited outstanding electrochemical properties, including high conductivity and catalytic activity. Previously, we developed a flexible 3D porous Ni_3_(HHTP)_2_ MOF/graphene-based sweat sensor with a highly reversible response to NH_4_^+^ ions [18]; however, a systematic stability study was not conducted. In this study, we also integrate the benefits of both MOF and rGO by designing a wearable sweat sensor based on a MOF/rGO composite. The rGO serves as a structure-directing agent [19], enhancing the growth and stability of the MOF, while also improving charge transfer kinetics due to its conductive properties. This synergistic combination enables the sensor to achieve high sensitivity and stability, making it ideal for the real-time, on-body monitoring of ammonium ions in sweat.

In this paper, we begin by detailing the preparation process of the key composite material and by performing comprehensive characterization, which demonstrates its effectiveness in modifying ISEs. We then fabricate it into a wearable sensor for ammonium ion detection, discussing the sensor’s sensitivity, LOD, and selectivity, along with measurements of its short-term and long-term stability, followed by preliminary on-body testing. The experimental results indicate that the MOF/rGO-based sensor exhibits high sensitivity for NH_4_^+^ detection, showing a Nernstian response of 59.2 ± 1.5 mV/log [NH_4_^+^] and a detection limit of 10^−6.37^ M. Notably, the sensor maintains stable measurements after seven days under ambient conditions. Therefore, preliminary testing suggests that our sensor is suitable for continuous ammonium monitoring during physical activity, presenting it as a promising candidate for wearable health monitoring applications.

## 2. Materials and Methods

### 2.1. Synthesis of Ni_3_HHTP_2_ MOF/rGO Composites

Graphene oxide (GO) was prepared using a modified Hummer’s method [20]. First, 2 g of high-purity graphite (Sigma-Aldrich, St. Louis, MO, USA) and 12 g of KMnO_4_ (Sigma-Aldrich, St. Louis, MO, USA) solid were mixed under magnetic stirring and cooled with ice to avoid excessive temperature. Concentrated H_3_PO_4_ (Sigma-Aldrich, St. Louis, MO, USA) and H_2_SO_4_ (Sigma-Aldrich, St. Louis, MO, USA) were mixed in a volume ratio of 7:1, and then slowly added to the above graphite mixture. Note that the solution temperature must not exceed 25 °C during the preparation of the solution. The mixture was then placed in an oil bath at 25 °C for 12 h to allow the reaction to proceed and was subsequently cooled to room temperature. Finally, a 10% H_2_O_2_ solution (Sigma-Aldrich, St. Louis, MO, USA) was added to the mixture, and it was washed several times with deionized water until settled. The resulting jelly like layer at the bottom was dialyzed for one week, yielding the foamed GO. For good dispersion, the prepared GO powder was sonicated in DI water for 6 h in advance.

**Figure 1:**
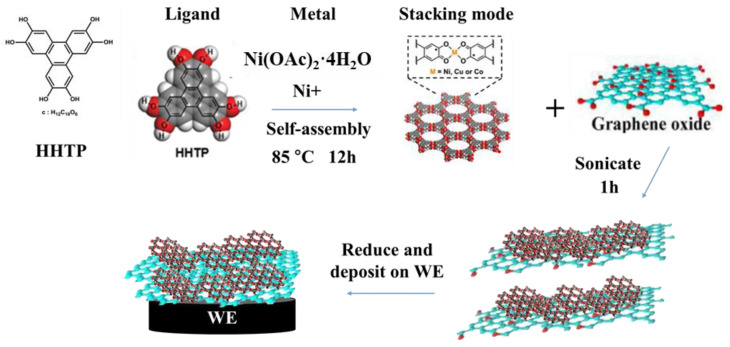
The synthesis of Ni-MOF/rGO composite and deposition on working electrode (WE).

To prepare the Ni_3_(HHTP)_2_ MOF, as shown in Figure 1, 7 mg of the linker HHTP (Sigma-Aldrich, St. Louis, MO, USA) and 10 mg of Ni(OAc)_2_·4H_2_O (Sigma-Aldrich, St. Louis, MO, USA) were mixed in 4 mL of DI water and dimethylformamide (DMF, Sigma-Aldrich, St. Louis, MO, USA) (v:v = 1:1) [21]. DMF was added as part of the modified solvent because it can promote the deprotonation reaction, which is beneficial for the formation of the MOF [22]. The mixture was heated for 12 h at 85 °C, and the resulting product was washed with DI water and acetone. After that, the Ni-MOF powders were dried under vacuum at 85 °C for 24 h.

To synthesize the Ni-MOF/rGO composite, various ratios of Ni_3_(HHTP)_2_ MOF and GO (ratios of 1:0, 20:1, 10:1, 5:1, 5:3, and 0:1 were applied in this study) were dissolved in 1 mL of DI water and sonicated for 1 h. Then, 1 μL of hydrazine solution was added (to reduce the GO to rGO) and the mixture was placed in an electric oven at 85 °C for 1 h. Subsequently, the mixture was washed with deionized water and acetone. The composite was dried under vacuum at 85 °C overnight and was then ready to be drop-cast onto the working electrode.

### 2.2. Fabrication of MOF/rGO-Modified Sweat Sensor

**Figure 2:**
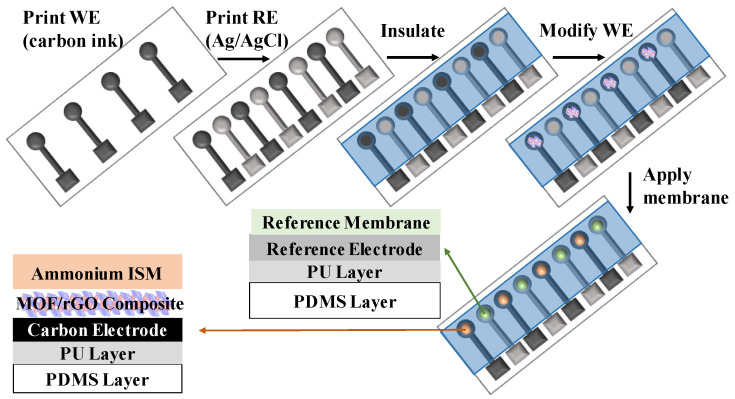
Schematic fabrication process of the all-solid-state MOF/rGO-modified wearable sweat sensor.

Figure 2 presents the fabrication steps of the MOF/rGO-modified wearable electrochemical sensors. First, to form a stretchable substrate, a poly-dimethylsiloxane (PDMS, Sigma-Aldrich, St. Louis, MO, USA) solution was placed onto a 2 mm thick glass plate. A vacuum chamber was used to prevent bubble formation in the PDMS, which was cured for 50 s at 120 °C. The PDMS was intended to be in a half-cured state to improve adhesion between the hydrophobic PDMS and the 150 µm thick hydrophilic polyurethane (PU, BASF, Mannheim, Germany) layer. The PU layer was formed by dissolving 15 wt% of PU in DMF solution, followed by curing at 50 °C for 30 min.

The reference electrode (RE) was fabricated by screen-printing a mixture of Ag/AgCl ink (Dupont, Wilmington, NC, USA) with 10 wt% PDMS onto the PU substrate and curing it at 120 °C for 6 min, achieving a thickness of 70 μm. The working electrode (WE) was prepared by screen-printing carbon ink (Zhongyi Inks Company, Zhongshan, China) with 10 wt% PDMS onto the PU substrate, also achieving a thickness of 70 μm. A PDMS insulating layer was then screen-printed and cured at 120 °C for 30 min on top of the WE and RE. The resulting cavity in the WE served as the active sensing area. Before drop-casting onto the WE, the pre-synthesized Ni-MOF/rGO composite was dissolved in 1 mL of DI water and sonicated for 1 h. Finally, the electrode was dried under N_2_ for 4 h.

The ammonium-selective membrane, constituting the ISM, was formed by drop-casting a solution onto the WE. This solution was composed of 1 mg of nonactin (Sigma-Aldrich, St. Louis, MO, USA), 66.8 mg of bis(2-ethylhexyl) sebacate (DOS, Sigma-Aldrich, St. Louis, MO, USA), and 32.2 mg of PU mixed in 1 mL of tetrahydrofuran (THF, Sigma-Aldrich, St. Louis, MO, USA). The drop-casting solution for the RE membrane was prepared by dissolving 78.1 mg of polyvinyl butyral (PVB, Sigma-Aldrich, St. Louis, MO, USA) and 50 mg of NaCl in 1 mL of methanol. To fabricate the stretchable ammonium-ion sensor, the RE membrane was formed by drop-casting 2 µL of the solution onto the Ag/AgCl electrode, while the WE was modified by drop-casting 2 µL of the ammonium-selective membrane solution. The sensor was left to dry overnight before use. 

## 3. Results

### 3.1. Morphology of 3D MOF/rGO Composite

**Figure 3:**
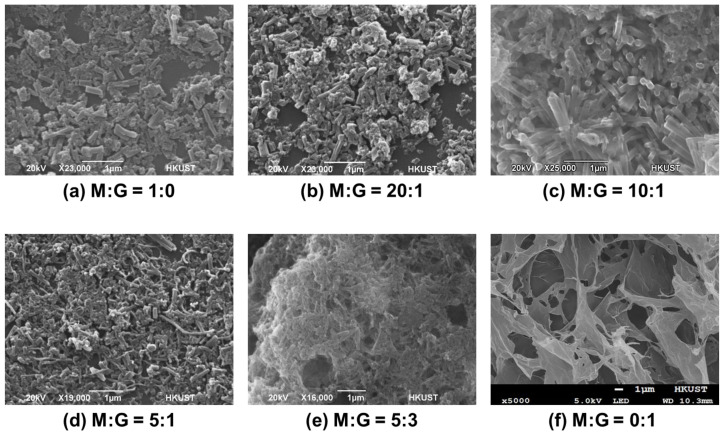
SEM micrographs of MOF/rGO composite structure at different MOF to rGO ratios (M:G).

To achieve highly porous 3D structures of the MOF/rGO composite, the morphology of the composites fabricated under a series of MOF/rGO ratios was compared. Representative Scanning Electron Microscope (SEM) micrographs of MOF/rGO composites with different MOF/rGO ratios are shown in Figure 3. When the MOF/rGO ratio is 0:1, Figure 3f shows the structure of rGO sheets only, with large pores. When the MOF/rGO ratio is 1:0, with no rGO present, the MOF crystals lie flat against the substrate surface without obvious 3D structures, as shown in Figure 3a.

The rGO can function as a structure-directing agent to control the growth of MOF, with oxygen groups on GO providing nucleation sites for MOF crystal binding [23]. Additionally, the oxygen atoms on either side of the MOF sheet can serve as structural guides in molecular assembly [24]. As a result, after mixing GO and MOF in varying ratios and conducting the reduction reaction, the morphology of the MOF/rGO composite exhibits distinct differences. For example, as shown in Figure 3b, at a MOF/rGO ratio of 20:1, the small amount of rGO leads to limited binding between MOF and rGO; at a MOF/rGO ratio of 10:1, the MOF crystals stand clearly upright (Figure 3c); for a MOF/rGO ratio of 5:1, the MOF crystals continue to bind to rGO while remaining upright (Figure 3d); with an increase in the MOF/rGO ratio to 5:3 (Figure 3e), the morphology increasingly resembles that of a 0:1 ratio, with graphene sheets being highly visible with a minimal presence of MOF crystals.

**Figure 4:**
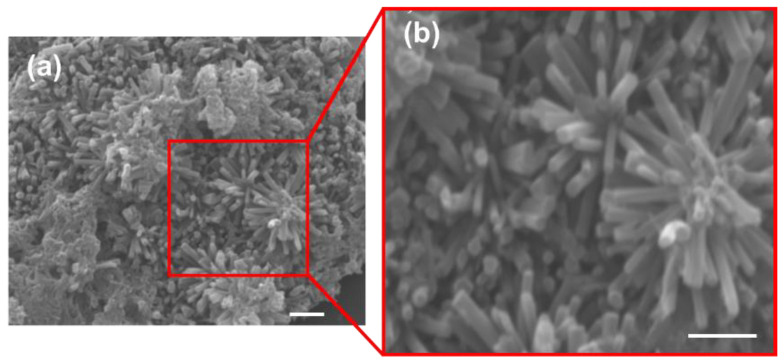
(**a**) SEM micrograph of the MOF/rGO composite (MOF/rGO ratio = 10:1); (**b**) zoomed-in SEM micrograph of the same MOF/rGO composite (All scale bars = 1 μm).

In summary, the morphological changes in the nanocomposite at different MOF/rGO ratios were influenced by the MOF/rGO binding mechanism and rGO agglomeration. The rGO served as a structure-directing agent, controlling MOF growth. In the absence of rGO, the MOF settled directly on the electrode surface. At low rGO content, MOF readily bound to rGO, forming freestanding MOF surfaces where oxygen functional groups on rGO facilitated MOF stacking. As rGO content increased, additional binding sites became available, leading to a more directionally aligned structure. An optimal MOF/rGO ratio of approximately 10:1 produced a distinctly aligned structure, enhancing electron transfer due to the increased surface area and abundance of active sites in the composite. However, as rGO content continued to increase, the MOF/rGO structure gradually flattened and eventually showed signs of agglomeration, as excess rGO tended to self-aggregate, reducing the effective interaction with MOF. Therefore, an optimized MOF/rGO ratio of 10:1 was used in subsequent experiments. Figure 4 shows the SEM micrographs of the highly porous 3D MOF/rGO composite (with the optimized MOF/rGO ratio of 10:1) formed on the WE surface. The rod-shaped crystallites were almost vertically aligned to the WE surface, which could facilitate the access of electrolyte and target analytes to the WE surface.

**Figure 5:**
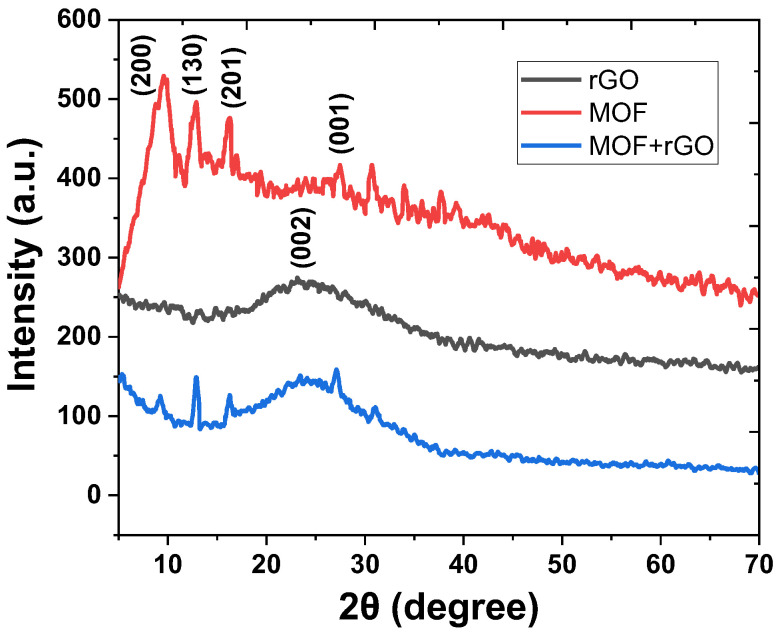
X-ray diffraction (XRD) patterns of rGO, MOF, and MOF/rGO composite.

The crystalline structures of rGO, MOF, and the MOF/rGO composite were further analyzed by X-ray diffraction (XRD). As shown in Figure 5, the XRD pattern of rGO (black curve) exhibited a broad peak near 2θ = 23.9°, corresponding to the (002) plane, which indicates a poor ordering of graphene sheets along the stacking direction [25]. For the Ni-based MOF, several low-angle peaks at 2θ = 9.2°, 13.1°, and 16.8° were observed in the XRD pattern (red curve), corresponding to the (200), (130), and (201) reflections, respectively [17]. Additionally, as the Ni-based MOF is a covalently connected layered material, an extra peak at 2θ = 27.6° (blue curve) indexed to the (001) reflection suggests π-π stacking between layers [26]. In the MOF/rGO composite, all these key characteristic peaks were retained, indicating that the presence of rGO does not alter the crystal structure of the Ni-based MOF in the composite.

### 3.2. Characterization of 3D MOF/rGO Composite

**Figure 6:**
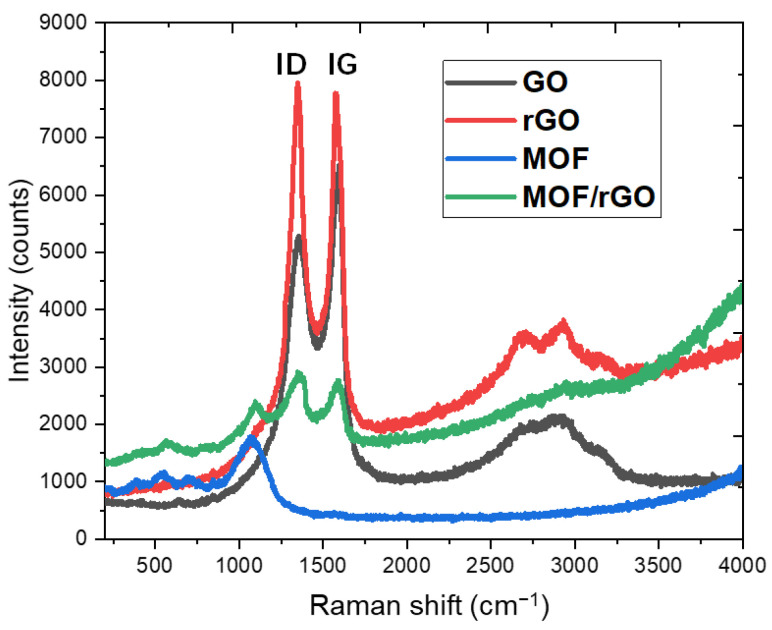
Raman spectra of GO, rGO, MOF, and MOF/rGO composite.

Raman spectroscopy (Renishaw 2000 micro-Raman Fourier transform spectrometer, Renishaw PLC, New Mills, England) was employed to further examine the structural characteristics of the GO, rGO, MOF, and rGO/MOF materials. As shown in Figure 6, the Raman spectra for both GO and rGO feature a D-band at 1348 cm^−1^, attributed to the sp^2^ hybridized carbon atom vibrations associated with graphene edges or structural disorder, and a G-band at 1591 cm^−1^, corresponding to the in-plane vibration of sp^2^ carbon atoms. Following the reduction in GO, a noticeable change was observed in the intensity ratio of the D-band (ID) to the G-band (IG), where GO showed ID < IG with an ID/IG ratio below 1 (~0.809). For rGO, this ID/IG ratio increased to approximately 1 (~1.025), while MOF/rGO exhibited the highest ID/IG ratio (~1.054), despite its lower overall intensity due to its porous structure. This increase in ID/IG was attributed to the reduction of oxygen-containing functional groups in GO and an increase in surface defects within the graphene, leading to topological disorder. A higher ID/IG ratio indicates a greater degree of defects, which enhances ion and electron conductance, thereby improving the cycling efficiency of the electrodes. Consequently, MOF/rGO was confirmed as a promising candidate for WE modification, offering superior ion transfer efficiency for sweat sensor applications.

In the MOF/rGO composites, all characteristic peaks appeared at their respective wavenumbers, confirming the coexistence of rGO and MOF within the composite. As shown in Figure 6, rGO exhibited higher Raman activity than MOF, likely due to its larger number of aromatic rings [27]. Additionally, the presence of 2D bands (~2600 to 2800 cm^−1^) was observed in both rGO and MOF/rGO composites. The 2D-to-G band ratio in the rGO layer was 0.2 ± 0.02, corresponding to an rGO thickness of approximately three to four layers [28]. This suggests that at a MOF-to-rGO ratio of 10:1, the rGO is well-exfoliated and interacts extensively with MOF crystals, without excessive accumulation (remaining at three to four layers). This optimal interaction results in a highly porous structure.

**Figure 7:**
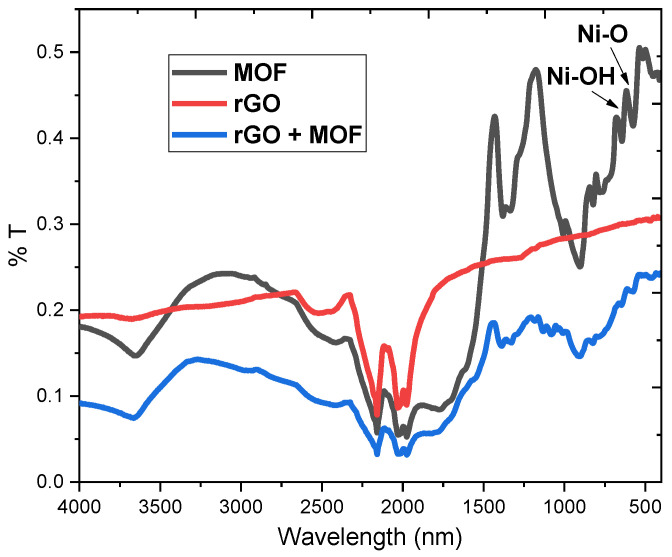
FTIR spectra of rGO, MOF, and rGO/MOF composite.

The Fourier transform infrared (FTIR) spectra of the rGO, MOF, and rGO/MOF powders are shown in Figure 7. After the reduction in graphene oxide, the absorption peak for -OH at 3212 cm^−1^ and the characteristic C=O absorption peak at 1720 cm^−1^ disappear, while the characteristic C=C absorption peak at 1673 cm^−1^ is significantly reduced, confirming the reduction in graphene oxide [29]. The bands at 627 and 691 cm^−1^ are attributed to Ni coordination with -O and -OH groups [30]. Meanwhile, most of the composite’s observable absorption bands in the FTIR spectrum correspond to the vibrational characteristics of the Ni-based MOF bonds, further demonstrating that MOF remains the dominant phase in the rGO/MOF composite and that its crystal structure remains unchanged.

### 3.3. Interface Properties of 3D MOF/rGO-Modified Electrodes

Electrochemical impedance spectra (EIS) were obtained using the AC impedance technique (CHI 660D, CH Instruments, Austin, TX, USA), as previously described [12]. The parameters for impedimetric detection were set as follows: an AC amplitude of ±100 mV and a frequency range of 0.1 Hz to 1 MHz.

**Figure 8:**
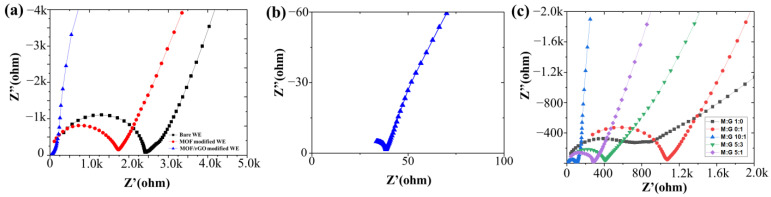
Nyquist plots of (**a**) bare WE, MOF-modified WE, and MOF/rGO-modified WE in 0.1 M NH_4_Cl solution (AC amplitude: 100 mV; frequency range: 0.1 Hz to 1 MHz); (**b**) magnified view of the Nyquist plot for the MOF/rGO-modified WE (MOF/rGO ratio of 10:1); (**c**) Nyquist plots of MOF/rGO-modified electrodes with various M:G ratios.

The Nyquist plots for the bare WE, MOF-modified electrode, and MOF/rGO-modified electrode are compared in Figure 8a. For the MOF/rGO-modified electrode, the Nyquist plot displays an approximate semi-circular trace at high frequencies, reflecting its charge transfer capability. The diameter of this semi-circle corresponds to the charge transfer resistance (*R*_ct_) of the WE. As shown in Figure 8b, the *R*_ct_ value for the MOF/rGO-modified electrode is lower than that of the MOF-modified and bare carbon electrodes. The EIS results were also fitted and verified by the equivalent circuit model (See Supplementary Materials Figure S1 and Table S2), in which the value of the *R*_ct_—with regard to the MOF/rGO-modified electrode (50.8 Ω)—was much reduced compared to that of the bare carbon electrode (2.41 × 10^3^ Ω). Additionally, at the low-frequency end, the Nyquist plot shows a steep linear profile, suggesting nearly ideal capacitive performance. Thus, the MOF/rGO composite, with its improved charge transfer rate and enhanced double-layer capacitance, is a compelling ion-to-electron transfer coating for the WE.

EIS was also performed on MOF/rGO-modified electrodes with varying MOF-to-rGO ratios (M:G ratio, as discussed in the SEM results). Figure 8c shows these EIS results, with Nyquist plots indicating that the MOF/rGO composite with a 10:1 M:G ratio exhibits the lowest *R*_ct_, as evidenced by the smallest semi-circle diameter, which corresponds to an enhanced charge transfer rate. Furthermore, the steep, nearly vertical linear profile following the semi-circle in the 10:1 M:G ratio plot suggests ideal capacitive behavior, indicative of enhanced double-layer capacitance. These findings confirm that the MOF/rGO composite with a 10:1 M:G ratio provides the highest charge transfer efficiency, making it a highly effective ion-to-electron transfer coating for the WE.

**Figure 9:**
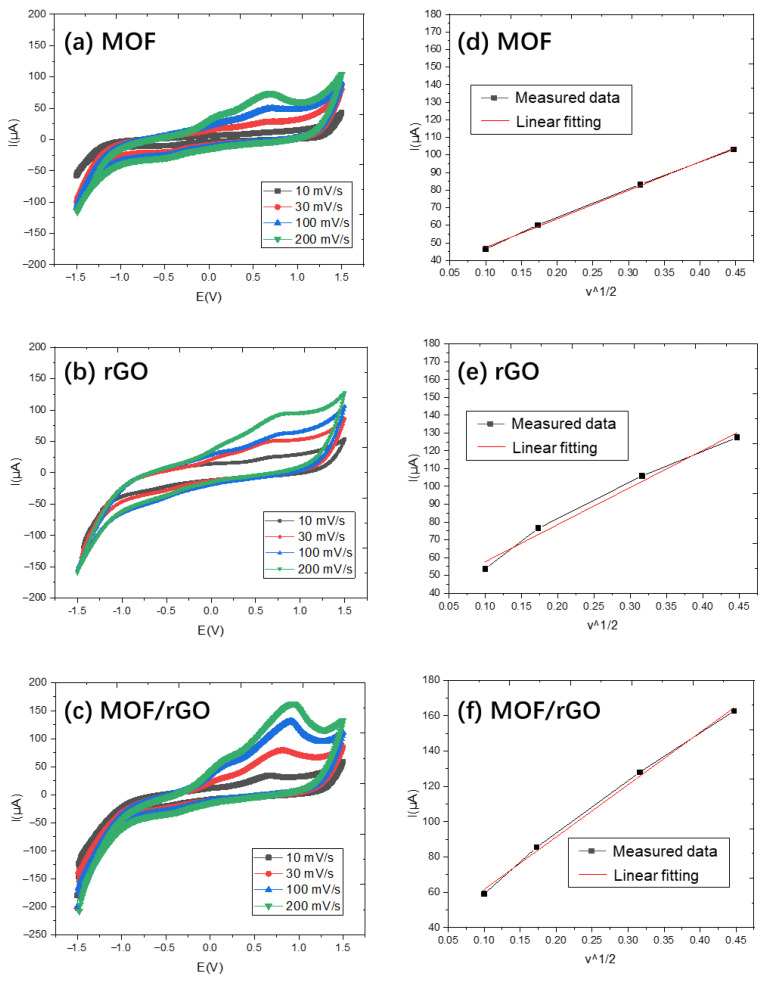
CV results at different scan rates for (**a**) MOF, (**b**) rGO, and (**c**) MOF/rGO-modified electrodes, and plots of anodic peak currents versus the square root of the scan rate for (**d**) MOF, (**e**) rGO, and (**f**) MOF/rGO.

To determine the effective electroactive area of MOF, rGO, and MOF/rGO-modified electrodes, cyclic voltammetry (CV) tests were conducted using the same setup as previously described [15]. Briefly, a three-electrode system was employed, consisting of the modified carbon electrode as the working electrode, platinum wire as the counter electrode, and an Ag/AgCl electrode as the reference electrode. The CV tests for MOF, rGO, and MOF/rGO-modified electrodes were performed in a 5.0 mM [Fe(CN)_6_]^3−^/^4−^ solution containing 0.1 M NH_4_Cl as the redox probe, with scan rates ranging from 10 to 200 mV·s^−1^. The results are shown in Figure 9a, Figure 9b and Figure 9c, respectively.

As the scan rate increased, the current values for all modified electrodes also increased. According to the Randles–Sevcik equation:

(1) $$I=2.69*10^{5}n^{3/2}v^{1/2}D^{1/2}AC$$

where *v* is the scan rate, *I* is the oxidation current derived from the CV curve, *n* is the number of electrons transferred, *D* is the diffusion coefficient, *A* is the electrode surface area, and *C* is the concentration.

Figure 9d, Figure 9e and Figure 9f plot the oxidation current *I* as a function of *v*^1/2^. Using the slope of the fitting curve, the effective electroactive areas of MOF-, rGO-, and MOF/rGO-modified electrodes were calculated as 0.21 cm^2^, 0.28 cm^2^, and 0.40 cm^2^, respectively. The effective surface area of the MOF/rGO-modified electrode was 1.9 times that of the MOF-modified electrode, attributable to the abundant MOF and rGO clusters on the WE surface, which provide more accessible active sites for enhanced ion transfer performance.

**Figure 10:**
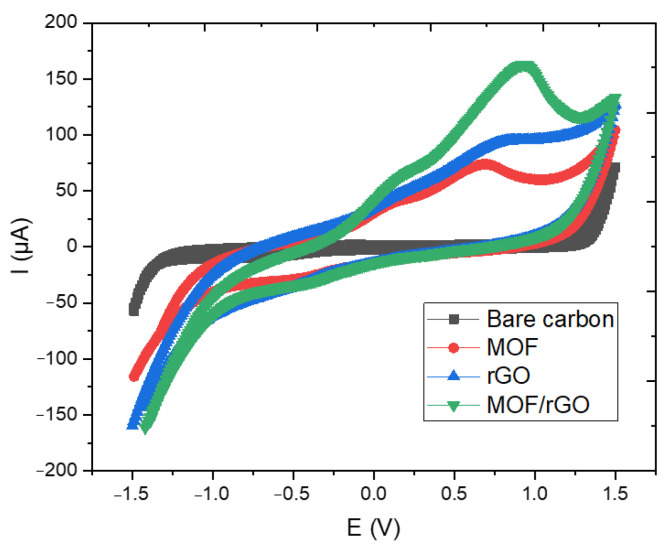
CV test results of the bare carbon electrode, MOF, rGO, and MOF/rGO-modified electrodes at a scan rate of 100 mV/s.

Figure 10 shows the CV test results of the bare carbon electrode, MOF, rGO, and MOF/rGO-modified electrodes at a scan rate of 100 mV/s in a 5.0 mM [Fe(CN)_6_]^3−^/^4−^ solution containing 0.1 M NH_4_Cl. The oxidation peak currents of MOF, rGO, and MOF/rGO exhibit a gradual increase compared to the bare carbon electrode, which can be attributed to the high conductivity of rGO and the composite materials, facilitating faster electron transfer kinetics. Additionally, the uniformly distributed MOF structures contribute to an increased effective surface area and enhanced electrical conductivity. This synergy between the two materials provides the modified WE with improved electrochemical performance.

Here is a brief summary of the material characterization results for the MOF/rGO composite: SEM, XRD, and FTIR analyses reveal that the MOF/rGO composite exhibits a highly porous 3D architecture, with the Ni-based MOF maintaining a stable crystal structure, as confirmed by Raman spectroscopy data. Furthermore, EIS measurements demonstrate an increased effective surface area, providing more accessible active sites that facilitate enhanced ion transfer. The combined Raman and EIS results provide compelling evidence that the MOF/rGO composite exhibits superior ion transfer efficiency, which could directly contribute to its improved performance in ammonium ion detection.

### 3.4. Ammonium Ion Detection

#### 3.4.1. Sensitivity, LOD and Slectivity

**Figure 11:**
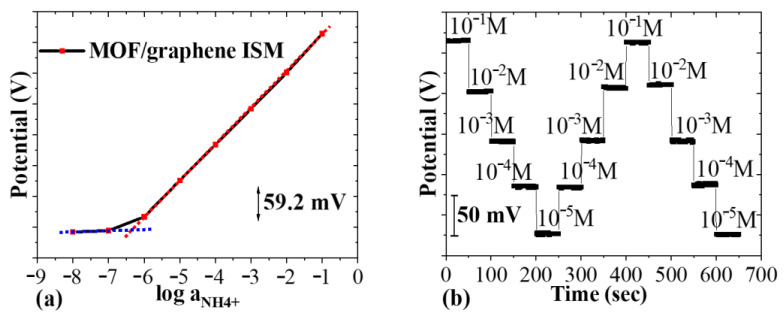
(**a**) Potential response of the sweat sensor to varying NH_4_^+^ concentrations over time (NH_4_Cl from 10^−8^ to 10^−1^ M); (**b**) reversibility test of the potential response (NH_4_Cl from 10^−5^ to 10^−1^ M).

The Open-Circuit Potential-Time (OCPT) technique (CHI 660D, CH Instruments, Austin, TX, USA) was employed to measure the potential difference as a function of time in a potentiometric test setup. During testing, the WE and RE were immersed in the same analyte solution, and the potential variation was recorded by OCPT with no current applied to the sensing system. NH_4_Cl solutions with ammonium concentrations ranging from 10^−8^ to 10^−1^ M were prepared. Between each test, the sensor chip was rinsed three times with DI water and dried before reuse. Figure 11a shows the potential output of the CHI660D analyzer between the WE and RE of the MOF/rGO-modified sensor as a function of ammonium concentration, demonstrating an almost ideal Nernstian response with a slope of 59.2 ± 1.5 mV/log [NH_4_^+^] (4.72% RSD, *N* = 4). From this response, a limit of detection (LOD) of 10^−6.37^ M was determined. Figure 11b shows the potential response over three cycles of NH_4_Cl testing solutions with concentrations ranging from 10^−5^ to 10^−1^ M, demonstrating consistent potentials at each concentration with minimal hysteresis. This indicates that the MOF/rGO-modified sensor exhibits good reversibility, a critical feature for wearable devices.

We use the Separate Solution Method (SSM) to theoretically estimate the selectivity of the sensor [31], as conducting experimental measurements is challenging at this stage. For the NH_4_^+^-selective electrode, K^+^ acts as an interfering ion. In sweat, typical K^+^ concentrations range from 0.2 to 6 mM, while NH_4_^+^ concentrations are between 0.1 and 1 mM. Given these concentration ranges, the selectivity coefficient becomes crucial for determining the sensor’s ability to distinguish NH_4_^+^ from K^+^ in real samples. The selectivity coefficient can be calculated theoretically, describing the sensor’s preference for NH_4_^+^ over K^+^, and is given by this equation:

(2) $$\log K_{{{NH}_{4}^{+},K}^{+}}^{pot}=\frac{\left(E_{K^{+}}-E_{{NH}_{4}^{+}}\right)z_{{NH}_{4}^{+}}F}{2.303RT}+(1-\frac{z_{{NH}_{4}^{+}}}{z_{K^{+}}})lga_{{NH}_{4}^{+}}$$

where *R* is the gas constant equal to 8.314510 J K^−1^mol^−1^, *T* is the absolute temperature equal to 298 K, *F* is the Faraday constant equal to 9.6485309 × 10^4^ C mol^−1^, $a_{{NH}_{4}^{+}}$ is the activity of NH_4_^+^, and $z_{{NH}_{4}^{+}}$ is the charge number.

The NH_4_^+^ selectivity coefficients of the nonactin-based sensor in the presence of K^+^ interference were reported to be in the range of −1.8 to −1.6 [32,33]. According to Equation (2), the NH_4_^+^ selectivity coefficients of the MOF/rGO-modified sensor were calculated to be slightly higher, ranging from −1.6 to −1.3. This difference is likely due to the unique distribution of functional groups and active sites in the MOF/rGO composite compared to nonactin, which affects its affinity for target and interfering ions [34], thus influencing the selectivity coefficient.

#### 3.4.2. Potential Stability

Potential stability, also known as short-term stability, is typically affected by electrode polarization in a constant measurement environment or by the presence of a water layer. Even in a stable measurement environment, electrode polarization can lead to potential drift. Ideally, the solid-contact layer should maintain a non-polarized interface with a high exchange current density, remaining unaffected by the small current input from the measuring amplifier. However, in practice, all solid-contact layers experience some degree of polarization. Potential drift, a critical criterion for stability, can be described by

(3) $$Potential\;drift=\frac{∆E}{∆t}=I/C$$

where *C* represents the low-frequency capacitance and *I* is the applied current.

Equation (3) highlights that a sufficiently large capacitance in the solid-contact layer is essential for achieving stable potential. Electrode potential stability can be further improved by using a solid-contact layer with high electrical double-layer capacitance. Electrode capacitance can be measured through chronopotentiometry, a method proposed by Bobacka [35]. In this method, at the start of potential recording, the electrode is polarized under a direct current (typically 1 nA). When the current direction is reversed, electrode resistance can be calculated based on the voltage drop, making this approach a standard for evaluating electrode stability.

**Figure 12:**
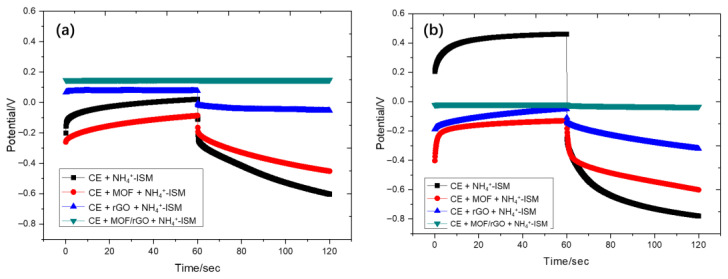
Chronopotentiometry test results of sensors with configurations of CE+NH_4_^+^ ISM, CE+MOF+NH_4_^+^ ISM, CE+rGO+NH_4_^+^ ISM, and CE+MOF/rGO+NH_4_^+^ ISM under currents of (**a**) ±1 nA and (**b**) ±10 nA.

Using this approach, chronopotentiometry tests were conducted on sensors with configurations of carbon electrode (CE)+NH_4_^+^ ISM, CE+MOF+NH_4_^+^ ISM, CE+rGO+NH_4_^+^ ISM, and CE+MOF/rGO+NH_4_^+^ ISM under currents of ±1 nA and ±10 nA. As shown in Figure 12, the test results indicate that the CE+MOF/rGO+NH_4_^+^ ISM configuration exhibits the most stable potential response under both current conditions, with minimal potential drift over time (7.2 µV/s (i = ±1 nA)) compared to other configurations. This enhanced stability is likely due to the MOF/rGO-modified layer’s higher double-layer capacitance, which effectively reduces potential drift. Moreover, the CE+rGO+NH_4_^+^ ISM configuration also shows relatively low potential drift, also likely due to the high electrical double-layer capacitance of rGO. This property makes rGO an effective component in minimizing potential drift, though it is not as effective as the combined MOF/rGO layer.

#### 3.4.3. Water Layer Test

**Figure 13:**
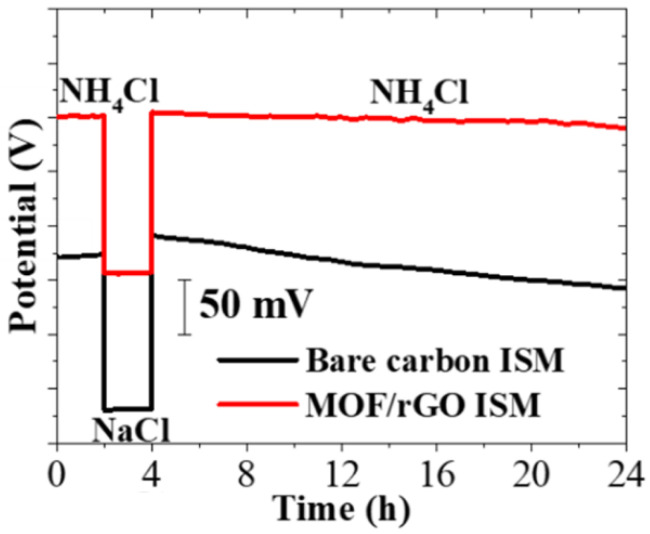
Aqueous layer test results for sensors with bare carbon ISM and MOF/rGO ISM in 0.1 M NH_4_Cl and 0.1 M NaCl solutions.

The formation of an aqueous layer between the sensing membrane and the electrode can adversely affect the potentiometric stability of the sensor. To investigate this, an additional aqueous layer test was conducted. The potential output of the sensor was first measured in a 0.1 M NH_4_Cl solution for 2 h, then in a 0.1 M NaCl solution for another 2 h, and finally returned to the 0.1 M NH_4_Cl solution for 20 h. As shown in Figure 13, a clear difference in potential response was observed between the bare carbon ISM and the MOF/rGO-modified electrode. The bare carbon ISM exhibited significant potential drift during the final 20 h period, whereas the MOF/rGO ISM maintained a stable potential response without drift. This result strongly indicates that the hydrophobic nature of the MOF/rGO layer helps to prevent water layer formation, thereby enhancing the potential stability of the sensor.

**Figure 14:**
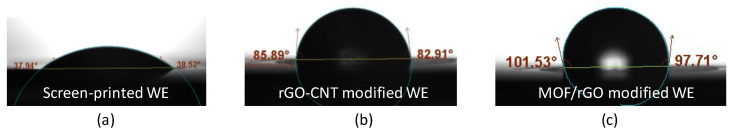
Contact angle test results of (**a**) screen-printed bare carbon WE, (**b**) rGO-CNT-modified WE, and (**c**) MOF/rGO-modified WE.

Figure 14 shows the contact angles of the screen-printed bare carbon WE (38.23°) and the MOF/rGO-modified WE (99.62°). These measurements confirm the hydrophobic nature of the MOF/rGO-modified WE, as its contact angle exceeds 90°. This hydrophobicity discourages water accumulation at the interface and is even greater than that observed in previously developed rGO-CNT-modified WEs [15]. These findings further support the results of the water layer test.

#### 3.4.4. Long-Term Stability

**Figure 15:**
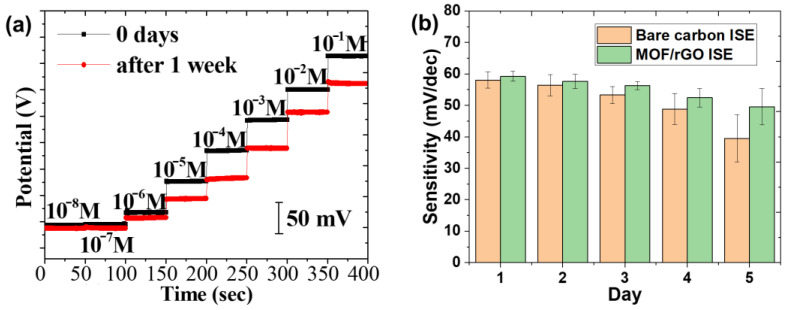
(**a**) Long-term stability of MOF/rGO-based sensors in NH_4_Cl solutions with electrolyte concentrations ranging from 10^−8^ to 10^−1^ M; (**b**) sensitivity change over time for sensors with bare carbon electrodes and MOF/rGO-modified electrodes.

To evaluate the long-term stability of the fabricated sensor, which is essential for daily use in wearable sensors, the sensor was initially tested in NH_4_Cl solutions with concentrations ranging from 10^−8^ to 10^−1^ M. It was then stored under ambient conditions for one week before being retested in NH_4_Cl solutions of the same concentrations. Figure 15a shows the comparison of the potential output as a function of NH_4_Cl concentration for both the fresh and aged sensors after 7 days. The output of the aged sensor remained stable and was still capable of accurately measuring NH_4_Cl concentrations from at least 10^−6^ to 10^−1^ M.

To further examine the sensitivity change in the sensor over time, three identical MOF/rGO sensors were tested for comparison. All tests were conducted in NH_4_Cl electrolyte solutions with concentrations ranging from 10^−^⁶ to 10^−1^ M as a benchmark for sensitivity calculation. Each result represents the average of three measurements, as shown in Figure 15b. The sensitivity of the MOF/rGO sensors stored under ambient conditions showed a gradual decrease over the first three days, with the sensitivity on the third day at 94.9% of the initial value. By the fourth day, the sensitivity declined to 88.3%. After five days, the sensors retained stable sensitivity, measuring 83.8% of the initial value and demonstrating that the MOF/rGO sensor has high stability and is suitable for use over at least five days.

### 3.5. On-Body Test

**Figure 16:**
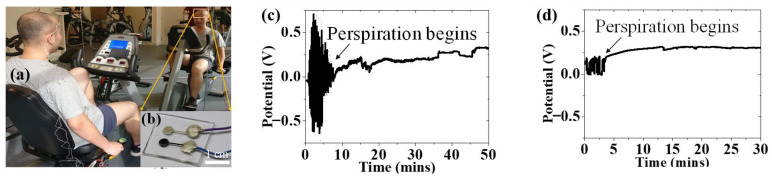
(**a**) On-body testing of the MOF/rGO-based sweat sensor placed on the participant’s forehead during exercise; (**b**) close-up view of the wearable MOF/rGO-modified sweat sensor; (**c**) and (**d**) real-time measurement of ammonium levels in sweat, showing the onset of perspiration and the subsequent stabilization of potential.

To evaluate the sensor’s stability and performance during physical activities, on-body tests were conducted under controlled conditions, where the subject performed light cycling exercises in a gym with an ambient temperature of approximately 24 °C. During the real-time monitoring of ammonium levels in sweat, the MOF/rGO-based sweat sensor was placed on the participant’s forehead, as illustrated in Figure 16a. Figure 16b shows a close-up of the wearable MOF/rGO-modified sweat sensor. This in situ measurement was also based on the OCPT technique to measure the potential difference as a function of time in the potentiometric test setup. The recorded potential signal was then converted into the NH_4_^+^ concentration over time using the sensor’s sensitivity of 59.2 mV/log [NH_4_^+^]. As shown in Figure 16c,d, the onset of perspiration was marked by a noticeable initial spike in potential, followed by a gradual stabilization as sweat levels increased. The sensor demonstrated stable ammonium ion detection, with consistent signal output despite initial fluctuations due to the onset of perspiration. This performance could be attributed to the hydrophobic nature of the MOF/rGO composite, which prevents water layer formation at the electrode interface, and its high double-layer capacitance, which minimizes potential drift caused by motion or sweat variability. Additionally, the optimized 3D porous structure of the composite could enhance charge transfer kinetics and increase the electroactive surface area, ensuring reliable performance under dynamic conditions.

The sensor recorded a stable ammonium concentration of approximately 4 × 10^−4^ M, consistent with typical ammonium levels found in human sweat. These results demonstrate the sensor’s effectiveness in detecting ammonium levels in sweat and highlight its potential for application as a wearable, real-time sweat monitoring device. Although the current measurement lasted only 50 min, long-term on-body testing will be conducted to evaluate the sensor’s ability to monitor ammonium concentration in sweat over extended periods. Furthermore, to develop a more robust sensor design, additional studies are necessary to fully understand the impact of interference from other sweat components, such as K^+^ and Na^+^, as well as the influence of environmental factors, including the subject’s physical activity and posture [36].

## 4. Conclusions

This study successfully developed a wearable, non-invasive ammonium sensor based on a MOF/rGO composite, demonstrating high potential for the real-time monitoring of NH_4_^+^ ions in sweat. The unique combination of the porous Ni-based MOF structure and the conductive properties of rGO provide enhanced charge transfer and increased electroactive surface area, resulting in a sensor with excellent sensitivity and stability. The MOF/rGO sensor achieved a Nernstian response of 59.2 ± 1.5 mV/log [NH_4_^+^] and a detection limit of 10^−6.37^ M, suitable for detecting low concentrations of ammonium ions in sweat. The sensor also exhibited robust potential stability, partly due to the hydrophobic nature of the MOF/rGO composite, which prevents the formation of a water layer between the sensing membrane and the electrode. On-body tests confirmed the practicality of the sensor for real-time sweat monitoring, with stable performance under prolonged conditions. As highlighted in Table S1, the MOF/rGO-based sensor exhibits superior performance compared to other reported ion-selective all-solid-state printed sensors based on nanocomposites for NH_4_^+^ detection. We anticipate that the MOF/rGO-based sensor will be a promising candidate for wearable health monitoring devices, with potential applications in assessing metabolic function and physical fitness.

For future work, several key areas could be explored to further enhance the potential of this sensor. First, it is essential to investigate strategies for minimizing interference from other components in sweat, such as K^+^, Na^+^, and Ca^2+^, through the development of more advanced ISM or composite materials. Additionally, understanding the effects of environmental factors, such as temperature and humidity, as well as physiological variables like physical activity, on sensor performance is crucial for ensuring its robustness in real-world applications. Long-term stability studies extending over several months would also be critical to assess the sensor’s reliability for continuous use in wearable devices, while the development of protective encapsulation techniques could improve its mechanical durability. Furthermore, integrating wireless data transmission capabilities for real-time, continuous monitoring would greatly enhance the sensor’s applicability in health monitoring systems [37]. Expanding on-body tests with a larger sample size will be essential to evaluate the sensor’s performance across different sweat compositions, ensuring its generalizability for broader use in wearable health technology. These directions open up several promising avenues for advancing the sensor’s capabilities and exploring its broader application in wearable health technology.

## Data Availability

The experimental data are contained within the article.

## Supplementary Materials

The following supporting information can be downloaded at: https://www.mdpi.com/article/10.3390/bios14120617/s1, Figure S1: Equivalent circuit model (ECM); Table S1: Comparison of the performance matrix of reported ion-selective all-solid-state printed sensors based on nanocomposites for NH₄⁺ detection; Table S2: Key parameters in ECM for EIS measurement; Table S3: Tools and consumables used in this manuscript [38,39].
