# Supplementary material for: A Highly Stable Electrochemical Sensor Based on a Metal–Organic Framework/Reduced Graphene Oxide Composite for Monitoring the Ammonium in Sweat
Source: Biosensors (Basel). 2024 Dec 15;14(12):617. doi: 10.3390/bios14120617 (PMC11674821; doi:10.3390/bios14120617)
Supplement: Supplementary file 1 [file biosensors-14-00617-s001.zip › biosensors-3335637-supplementary.pdf]

1. The electrochemical mechanism of NH_4^+ detection and its comparison with that of met-al-oxide thin film pH sensors

Ammonium ion-selective electrodes (NH_4^+-ISE) are electrochemical sensors that convert the activity of the selected ion, NH_4^+, in a sample solution into a potential output. The sensor consists of a thin ion-selective membrane containing an ionophore, a common matrix polymer (e.g., PVC), a plasticizer (e.g., NOPE, DOS), and an ionic site. The NH_4^+-selective membrane is doped with ionophores that form reversible complexes with NH_4^+ ions. These ionophores, which are lipophilic and strongly bind to NH_4^+ ions, provide the necessary selectivity of the sensor. The sensor works by allowing NH_4^+ ions to move from a high concentration to a low concentration end, creating a potential difference proportional to the ion activity. The ionophore's molecular structure ensures selective binding to NH_4^+, as it has a much larger free energy of complexation with NH_4^+ compared to other ions [1], providing high specificity.

In contrast, the sensing mechanism of MOx-based pH sensors relies on the interaction between the metal-oxide surface and protons (H^+) or hydroxide ions (OH^-) from the solution. This interaction leads to the formation of surface hydroxyl groups, and the resulting changes in the surface potential create a potential difference that is proportional to the pH of the solution [2]. MOx sensors rely on ion exchange and the creation of an electrical double layer (EDL) at the sensor-solution interface, where the charged surface groups generate a potential that can be measured.

While the sensing mechanisms of NH_4^+-ISEs and MOx-based pH sensors are fundamentally different, both rely on electrochemical principles where surface interactions with ions (NH_4^+ for the ISE and H^+/OH^- for the MOx sensors) lead to changes in the potential that can be measured. However, NH_4^+-ISEs provide a more specific detection of ammonium ions by using selective ionophores, while MOx-based pH sensors measure the overall hydrogen ion activity and provide a broader measure of acidity or alkalinity.

2. Comparison of the performance matrix

Table S1. Comparison of the performance matrix of reported ion-selective all-solid-state printed sensors based on nanocomposites for NH_4^+ detection.

Ionophore	Solid Contact	Sensitiviy/ mV/log [NH_4^+]	LOD/M	Linearity Range/M	Potential Stability	Long-term Stability	Reference
SiO2/ZrO2/Phosphate-NH_4^+	graphite powder	31.3	1.6×10^{-7}	7.7×10^{-7}–4.0×10^{-2}	NA	NA	[3]
nonactin-based	CNT-PVC composites[1]<br>MMA-DMA copolymers[2]	50.9<br>50.7	2.6×10^{-7}<br>2.2×10^{-7}	1.0×10^{-6}–1.0×10^{-3}	< 1,000 μV/h (i=0)<br>3,600 μV/h (i=0)	NA	[4]
nonactin-based	CPANI[3]	54.2	1.0×10^{-6}	1.0×10^{-4}–1.0×10^{-1}	NA	NA	[5]
nonactin-based	graphite–PVB[4]	57.3	4.8×10^{-6}	1.0×10^{-5}–1.0×10^{-1}	NA	NA	[6]
nonactin-based	3D graphene–CNT[5]	59.6	1.0×10^{-6}	1.0×10^{-6}–1.0×10^{-1}	NA	NA	[7]
nonactin-based	3D Ni_3HHTP_2 MOF/rGO Composites	59.2 ± 1.5	$1.0 \times 10^{-6.37}$	10^{-6} to 10^{-1}	7.2 μV/s (i = ± 1 nA)	7 days	This work

[1] composite polyvinyl chloride membrane impregnated with carbon nanotubes; [2] plasticizer-free methyl methacrylate–decyl methacrylate copolymer; [3] copolymer of aniline/2,5-dimethoxyaniline; [4] graphite particles embedded in a polyvinyl butyral matrix; [5] 3D graphene oxide–carbon nanotubes composite.

3. The parameters derived from the ECM for bare working electrode, MOF-modified electrode, and MOF/rGO-modified electrode.

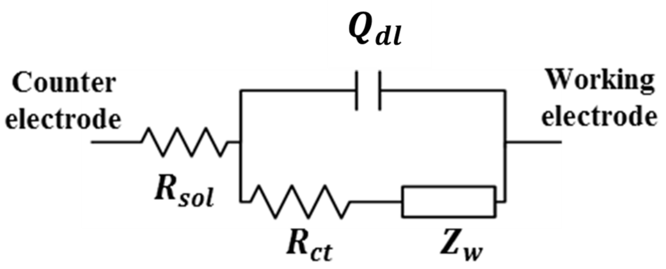

Figure S1. Equivalent circuit model (ECM).

Table S2. Key parameters in ECM for EIS measurement.

Electrode	R_{sol}(Ω)	R_{ct}(Ω)	Z_W (Ω·$s^{-1/2}$)	Q_{dl}(S·s^n)	n
**Bare working electrode**	189.7	2.41×10^3	3.5×10^4	1.59×10^{-7}	0.802
**MOF-modified electrode**	268.1	1.73×10^3	2.2×10^4	3.34×10^{-7}	0.867
**MOF/rGO-modified electrode**	146.4	50.8	2.3×10^3	4.23×10^{-7}	0.824

R_{sol}: **Solution resistance**, which is unaffected by the target ion
R_{ct}: **Charge transfer resistance**, which is influenced by the energy potential with the complex ion transfer, and it affects the electrostatic and steric energy barrier with the ion passing
Z_W: **Warburg impedance**, which stands for the impedance at low frequencies and it governs the delay arising from the diffusion of redox species to the electrode
Q_{dl}: **Nonideal electrochemical double layer capacitance**, which describes the quantity of charge formed in the electrical double layer
n: index, which refelects the perfection of Q_{dl}

4. Table S3. Tools and consumables used in this manuscript.

Tool/Consumable	Make/Model	Country of Origin
Electrochemical Analyzer	CHI 660D	USA
Graphene Oxide (GO)	Sigma-Aldrich, MO	USA
Metal-Organic Framework(Nis(HHTP)$_2$)	CD Bioparticles, NY	USA
Poly(dimethylsiloxane)(PDMS)	Sigma-Aldrich, MO	USA
Polyurethane(PU)	BASF, Mannheim	Germany
Silver/Silver Chloride Ink (Ag/AgCl)	Dupont, Wilmington, NC, USA	USA
Carbon lnk	Zhongyi Inks Company, Zhongshan,China	China
Bis(2-ethylhexyl) Sebacate (DOS)	Sigma-Aldrich, MO	USA
Nonactin	Sigma-Aldrich, MO	USA
Polyvinyl Butyral (PVB)	Sigma-Aldrich, MO	USA
Tetrahydrofuran (THF)	Sigma-Aldrich, MO	USA

Reference:

1. Suzuki, Koji, Dwi Siswanta, Takeshi Otsuka, Tsuyoshi Amano, Takafumi Ikeda, Hideaki Hisamoto, Ryoko Yoshihara, and Shigeru Ohba. "Design and Synthesis of a More Highly Selective Ammonium Ionophore Than Nonactin and Its Application as an Ion-Sensing Component for an Ion-Selective Electrode." Analytical Chemistry 72, no. 10 (2000): 2200-05.
2. Manjakkal, Libu, Dorota Szwagierczak, and Ravinder Dahiya. "Metal Oxides Based Electrochemical Ph Sensors: Current Progress and Future Perspectives." Progress in Materials Science 109 (2020): 100635.
3. Coutinho, Cláudia FB, Alfredo A Muxel, Crystian G Rocha, Débora A de Jesus, Rení VS Alfaya, Flaveli AS Almeida, Yoshitaka Gushikem, and Antonio AS %J Journal of the Brazilian Chemical Society Alfaya. "Ammonium Ion Sensor Based on Sio2/Zro2/Phosphate-Nh4+ Composite for Quantification of Ammonium Ions in Natural Waters." 18 (2007): 189-94.
4. Athavale, Rohini, Ilga Kokorite, Christian Dinkel, Eric Bakker, Bernhard Wehrli, Gastón A. Crespo, and Andreas Brand. "In Situ Ammonium Profiling Using Solid-Contact Ion-Selective Electrodes in Eutrophic Lakes." Analytical Chemistry 87, no. 24 (2015): 11990-97.
5. Huang, Yuanfeng, Jun Li, Tianya Yin, Jianjun Jia, Qian Ding, Hao Zheng, Chen-Tung Arthur Chen, and Ying Ye. "A Novel All-Solid-State Ammonium Electrode with Polyaniline and Copolymer of Aniline/2,5-Dimethoxyaniline as Transducers." Journal of Electroanalytical Chemistry 741 (2015): 87-92.
6. Ivanišević, Irena, Stjepan Milardović, Antonia Ressler, and Petar Kassal. "Fabrication of an All-Solid-State Ammonium Paper Electrode Using a Graphite-Polyvinyl Butyral Transducer Layer." Chemosensors, no. 12 (2021).
7. Hua, Yunzhi, Mingxiang Guan, Linzhong Xia, Yu Chen, Junhao Mai, Cong Zhao, and Changrui Liao. "Highly Stretchable and Robust Electrochemical Sensor Based on 3d Graphene Oxide–Cnt Composite for Detecting Ammonium in Sweat." 13, no. 3 (2023): 409.
